# Comparison of the reliability of corneal curvature and eccentricity measurements in myopic eyes using four different devices

**DOI:** 10.3389/fmed.2025.1519992

**Published:** 2025-04-04

**Authors:** Dongyi Qu, Jia Yu, YueHua Zhou

**Affiliations:** ^1^Beijing Aier Eye Hospital, Beijing, China; ^2^Ineye Hospital of Chengdu University of TCM, Chengdu, Sichuan, China; ^3^Key Laboratory of Sichuan Province Ophthalmopathy Prevention and Cure and Visual Function Protection, Chengdu, Sichuan, China; ^4^Beijing Ming Vision and Ophthalmology, Beijing, China; ^5^Eye School of Chengdu University of TCM, Chengdu, Sichuan, China

**Keywords:** corneal curvature, eccentricity, RK-F1, TMS-4, Medmont, Pentacam

## Abstract

Myopia is a global public issue which is increasing worldwidely. Instruments are essential for measurement in the diagnosis and evaluation of myopia. Comparing the agreement of corneal curvature and eccentricity measurements obtained using four different devices is meaningful for clinical research. We present enrolled 175 patients in this prospective study. The corneal curvature were measured by The Canon RK-F1, Tomey TMS-4, Medmont E300, Pentacam HR, eccentricity measured by last three devices. The agreement and differences were compared among the four instruments. There was a weak correlation between the TMS-4 and Pentacam HR in eccentricity steep (Es) compared to other devices. The Bland–Altman plots with 95% level of agreement showed low agreement of corneal curvature measured by the four instruments. The 95% LoA of K steep (Ks) and K flat (Kf) were > 0.5D for all instruments. For eccentricity, eccentricity mink (Em), also eccentricity flat, showed high agreement among the TMS-4, Pentacam HR, and Medmont E300 topographers, but Es showed low agreement. The agreement of corneal curvature measured by the four instruments was low, which cannot be used interchangeably in clinical practice. The Es obtained from TMS-4, Medmont E300, and Pentacam HR can be used interchangeably.

## Introduction

The measurement of refractive error, corneal curvature, and corneal eccentricity in children with high accuracy and repeatability is important in vision screening, clinical evaluation, and research. Reproducible keratometry is particularly important for performing cataract and refractive surgery, and for diagnosing corneal diseases such as keratoconus and monitoring changes in corneal curvature over time. Steep keratometry, flat keratometry, and corneal eccentricity are must-have data to evaluate corneal central and peripheral shape and conduct orthokeratology lens fitting. Orthokeratology is widely used to control myopia progression in children. There is a high prevalence of myopia in China, especially in recent years, and orthokeratology is performed in most ophthalmological departments (23–25). Corneal topography is widely used to measure the corneal shape. A variety of instruments can be used for corneal curvature and cornea eccentricity measurements. The Canon RK-F1 is an autorefractor keratometer that performs the automatic measurement of both corneal curvature and refractive power.

Tomey TMS-4 is a corneal topographer based on the principle of the Placido disc. This device is equipped with a replaceable 25-ring and 31-ring small cone-disc Placido imaging device and can obtain images composed of up to 7,936 points in less than 3 s when combined with the computer technology of the color topographic map analysis system, resulting in the acquisition of clear and detailed corneal information (1, 22). The acquired data is processed by a computer and displayed through different topographic map modes. Medmont E300 is a Placido disc cone-based computerized videokeratometer, which draws the topographic map of the human corneal surface with the Placido ring. Tomey and medmont are both based on the Placido disc principle. An alternative method is corneal tomography by Pentacam. Pentacam HR (software version 1.2r43) is a non-contact anterior segment analyzer with a rotating Scheimpflug camera system. These instruments vary in accuracy and repeatability. Quantifying the differences between instruments will be helpful for clinical diagnosis, especially for orthokeratology fitting. Orthokeratology trial lens parameters depend on the corneal data. A small difference originated from different instruments will affect the trial lens parameters choices.

To our knowledge, no study has reported differences in measurements among four devices and evaluated the interchangeability and agreement of eccentricity obtained using topographer (TMS-4 and Medmont E300) and anterior segment analyzer (Pentacam HR). The main purpose of this study was to assess the agreement of corneal curvature and eccentricity measurements obtained from four different devices. This information may provide a valuable standard for optometrists, against which they can choose the first trial lens efficiently and design their Ortho-K lens prescription accurately.

## Subjects and methods

### Patients

In this study, 175 patients aged 7–15 years enrolled for Orthokeratology (OK) lens fitting from January to June 2021 were selected. Informed consent was provided according to the Declaration of Helsinki. The inclusion criteria were as follows: (1) Best corrected distance visual acuity (BCVA) ≥ 20/25; (2) No history of refractive surgery and contact lens; (3) No active eye disease; (4) Stable tear film, Schirmer’s test results >5.0 mm, and tear film break-up time > 5 s. All subjects underwent a full ophthalmic examination. Corneal curvature and eccentricity were obtained by a qualified and skilled examiner on the same day using the instruments presented above in random order.

### Instruments

The Canon RK-F1.

Tomey TMS-4 (software version 22C-200S-2A5).

The Medmont E300 (software version 6.2.7.1).

Pentacam HR (software version 1.2r43).

### Measurement

All the eyes were grouped into the moderate myopia group with refraction > −3.00D, medium myopia group > −6.00D and < −3.00D, and high myopia group < −6.00D.

Based on the purpose of this study, the corneal curvature was measured on a 3-mm diameter field of the central cornea, and the eccentricity was measured on a 9-mm diameter field of the cornea. Measurements were performed by four qualified and skilled examiners and completed between 9 and 12 a.m. on the same day to minimize the influence of the circadian rhythm on the results. Checking sequence was random. The equipments were calibrated according to the manufacturer’s instructions regularly. If necessary, imaging was repeated until quality acceptable. Only the right eye of each candidate was selected. If the image obtained from the right eye was disqualified the left eye was selected. Candidates were excluded from the study when measurements obtained from both eyes were disqualified. In Canon RK-F1, the measurements without the “*” mark were considered to be available. In TMS-4, the best quality image with the highest score was chosen for analysis. In Medmont E300, each image was scored, and the image with the highest score was selected (at least >75 points). Pentacam HR displayed the image quality automatically through the Examination Quality Specification system, and the images with “OK” were analyzed.

### Statistical analysis

Statistical analysis was conducted using SPSS software for Windows version 26. The distributions of all collected data were tested for normality using Kolmogorov–Smirnov tests prior to statistical analysis. All quantitative data with a normal distribution are represented as mean ± standard deviation (mean ± SD). The paired t-test was used to compare the measurements of different instruments, and Pearson correlation analysis was used to assess the correlation of measurements with various instruments (Spearman correlation analysis was selected when the normality distribution was not met). A *p* value <0.05 was considered statistically significant. Bland–Altman plots were used to assess the agreement between the devices, showing the mean and 95% limits of agreement LoA (Level of Agreement), calculated as: mean ± 1.96 standard deviation. The narrower the interval between the 95% LoA, the higher the consistency between the instruments.

## Results

Overall, 175 eyes (170 right eyes) from 175 OK lens fitting candidates were recruited, including 63 males (mean age 10.27 ± 1.99 years) and 112 females (mean age 10.26 ± 1.99 years). Refractive power ranged from −0.75D to −5.75D (spherical power was −2.42 ± 1.26D, astigmatism power − 0.55 ± 0.56D, and spherical equivalent −2.69 ± 1.36D) and was measured after cycloplegia.

The Ks was 44.06 ± 1.48D, 44.21 ± 1.47D, 44.27 ± 1.47D, and 43.78 ± 1.43D (*F* = 4.225, *p* < 0.05) measured by RK-F1 autorefractor, TMS-4 topographer, Medmont E300 topographer, and Pentacam HR, respectively.

Tables 1, 2 show the Ks and Kf values according to gender and spherical equivalent power. There was no statistically significant difference in Ks and Kf values among the four devices in the male group, but a statistically significant difference was found in the female group (Ks: *F* = 2.805, Kf: *F* = 3.044, all *p* < 0.05). In the mild myopia group, the Kf value was insignificant, whereas it was statistically significant in the moderate myopia group (*F* = 3.044, *p* < 0.05).

**Table 1:** Ks distance by gender and spherical equivalent (mean ± SD, D).

	Canon RK-F1	TMS-4	Medmont E300	Pentacam	*N*	*F*	*P*
Overall	44.06 ± 1.48	44.21 ± 1.47	44.27 ± 1.47	43.78 ± 1.43	175	3.832	<0.05
Male	43.39 ± 1.39	43.51 ± 1.36	43.61 ± 1.35	43.13 ± 1.32	63	1.525	0.21
Female	44.44 ± 1.39	44.59 ± 1.39	44.64 ± 1.41	44.15 ± 1.37	112	2.805	<0.05
−0.75D < SE ≤ −3.00D	43.94 ± 1.50	44.09 ± 1.48	44.15 ± 1.49	43.68 ± 1.46	109	2.246	0.08
−3.00D<SE ≤ −6.00D	44.35 ± 0.43	44.48 ± 1.42	44.55 ± 1.42	44.04 ± 1.38	61	2.805	<0.05

SD, standard deviation; Ks, steep corneal curvature; D, diopter; F, repeated-measures analysis of variance test.

**Table 2:** Kf distance by gender and spherical equivalent (mean ± SD, D).

	Canon RK-F1	TMS-4	Medmont E300	Pentacam	*N*	*F*	*P*
Overall	42.67 ± 1.33	42.93 ± 1.35	42.90 ± 1.32	42.48 ± 1.33	175	4.26	<0.05
Male	42.01 ± 1.19	42.27 ± 1.21	42.25 ± 1.16	41.82 ± 1.19	63	2.05	0.11
Female	43.04 ± 1.27	43.30 ± 1.29	43.27 ± 1.27	42.85 ± 1.26	112	3.04	<0.05
−0.75D < SE ≤ −3.00D	42.59 ± 1.32	42.84 ± 1.35	42.81 ± 1.31	42.39 ± 1.31	109	2.74	<0.05
−3.00D<SE ≤ −6.00D	42.88 ± 1.35	43.14 ± 1.34	43.11 ± 1.34	42.69 ± 1.35	61	3.04	<0.05

SD, standard deviation; Kf, flat corneal curvature; D, diopter; F, repeated-measures analysis of variance test.

Tables 3, 4 show the Es and Em according to gender and spherical equivalent power. Es was 0.57 ± 0.14, 0.43 ± 0.17, and 0.64 ± 0.11 measured by TMS-4 topographer, Medmont E300 topographer, and Pentacam HR, respectively (*x*^2^ = 135.03, *p* < 0.05). Em was 0.52 ± 0.10, 0.63 ± 0.08, and 0.55 ± 0.08 measured by TMS-4 topographer, Medmont E300 topographer, and Pentacam HR, respectively (*x*^2^ = 160.80, *p* < 0.05). The measurements of Es and Em in different gender and spherical equivalent groups were statistically significant (all *p* < 0.05).

**Table 3:** Es distance by gender and spherical equivalent.

	TMS-4	Medmont E300 s	Pentacam	*N*	χ^2^	*P*
Overall	0.57 ± 0.14	0.43 ± 0.17	0.64 ± 0.11	175	135.03	<0.05
Male	0.59 ± 0.12	0.42 ± 0.17	0.65 ± 0.11	63	72.08	<0.05
Female	0.56 ± 0.15	0.43 ± 0.17	0.63 ± 0.11	112	90.58	<0.05
−0.75S < SE ≤ −3.00D	0.58 ± 0.14	0.43 ± 0.17	0.64 ± 0.12	109	97.86	<0.05
−3.00D<SE ≤ −6.00D	0.57 ± 0.15	0.43 ± 0.16	0.64 ± 0.10	61	61.45	<0.05

SD, standard deviation; Es, steep meridians eccentricity, χ^2^, Kruskal-Wallis H test.

**Table 4:** Em distance by gender and spherical equivalent.

	TMS-4	Medmont E300	Pentacam	*N*	χ^2^	*P*
Overall	0.52 ± 0.10	0.63 ± 0.08	0.55 ± 0.08	175	160.80	<0.05
Male	0.52 ± 0.11	0.63 ± 0.09	0.56 ± 0.09	63	41.62	<0.05
Female	0.52 ± 0.10	0.63 ± 0.07	0.55 ± 0.08	112	94.69	<0.05
−0.75S < SE ≤ −3.00D	0.52 ± 0.11	0.63 ± 0.08	0.55 ± 0.09	109	77.20	<0.05
−3.00D<SE ≤ −6.00D	0.52 ± 0.09	0.64 ± 0.07	0.56 ± 0.08	61	53.89	<0.05

SD, standard deviation; Es, steep meridians eccentricity; χ^2^, Kruskal-Wallis H test.

### Correlation

A strong positive correlation was found in the comparison of Ks and Kf between any two instruments (*r* = 0.983 ~ 0.996, *p* < 0.01). Comparing the results for Ks, the correlation was the strongest between RK-F1 and TMS-4 (*r* = 0.992, *p* < 0.01, Table 5) and the weakest between TMS-4 and Medmont E300 (*r* = 0.983, *p* < 0.01). Comparing the results for Kf, a similar outcome was found; the correlation was the strongest between RK-F1 and TMS-4 (*r* = 0.996, *p* < 0.01) and weakest between RK-F1 and Medmont E300 (*r* = 0.989, *p* < 0.01).

**Table 5:** Correlation and difference of corneal curvature and eccentricity measured by four instruments.

Devices		Correlation		Difference			
		*r*	*p*	Mean difference (mean ± SD)	*t*	*p*	95%LoA
RK-F1 vs. TMS-4	Ks	0.992	<0.01	0.14 ± 0.19	9.96	<0.01	−0.51 ~ 0.23
	Kf	0.996	<0.01	0.26 ± 0.12	27.48	<0.01	−0.50 ~ −0.02
RK-F1 vs. Medmont E300	Ks	0.985	<0.01	0.21 ± 0.25	10.77	<0.01	−0.70 ~ 0.29
	Kf	0.989	<0.01	0.23 ± 0.20	15.35	<0.01	−0.62 ~ 0.16
RK-F1 vs. Pentacm	Ks	0.990	<0.01	−0.28 ± 0.21	−18.12	<0.01	−0.12 ~ 0.67
	kf	0.995	<0.01	−0.19 ± 0.13	−18.76	<0.01	−0.07 ~ 0.45
TMS-4 vs. Medmont E300	Ks	0.983	<0.01	0.06 ± 0.27	3.15	<0.01	−0.59 ~ 0.46
	Kf	0.990	<0.01	−0.03 ± 0.19	−2.08	0.039	−0.35 ~ 0.40
	Es	0.444*	<0.01	−0.14 ± 0.19	−9.86	<0.01	−0.22 ~ 0.50
	Em	0.716*	<0.01	0.12 ± 0.09	17.71	<0.01	−0.28 ~ 0.05
TMS-4 vs. Pentacam	Ks	0.990	<0.01	−0.42 ± 0.20	−27.50	<0.01	0.02 ~ 0.83
	Kf	0.996	<0.01	−0.45 ± 0.13	−46.70	<0.01	0.20 ~ 0.70
	Es	0.579*	<0.01	0.07 ± 0.14	6.53	<0.01	−0.34 ~ 0.20
	Em	0.786*	<0.01	0.04 ± 0.08	5.82	<0.01	−0.19 ~ 0.12
Medmont E300 vs. Pentacam	Ks	0.990	<0.01	−0.49 ± 0.21	−31.05	<0.01	0.08 ~ 0.89
	Kf	0.991	<0.01	−0.42 ± 0.18	−30.48	<0.01	0.06 ~ 0.78
	Es	0.341*	<0.01	0.21 ± 0.17	16.62	<0.01	−0.28 ~ 0.05
	Em	0.838*	<0.01	−0.08 ± 0.05	−22.6	<0.01	−0.01 ~ 0.17

The data marked with “*” indicates using Spearman correlation analysis.

**Figure 1:**
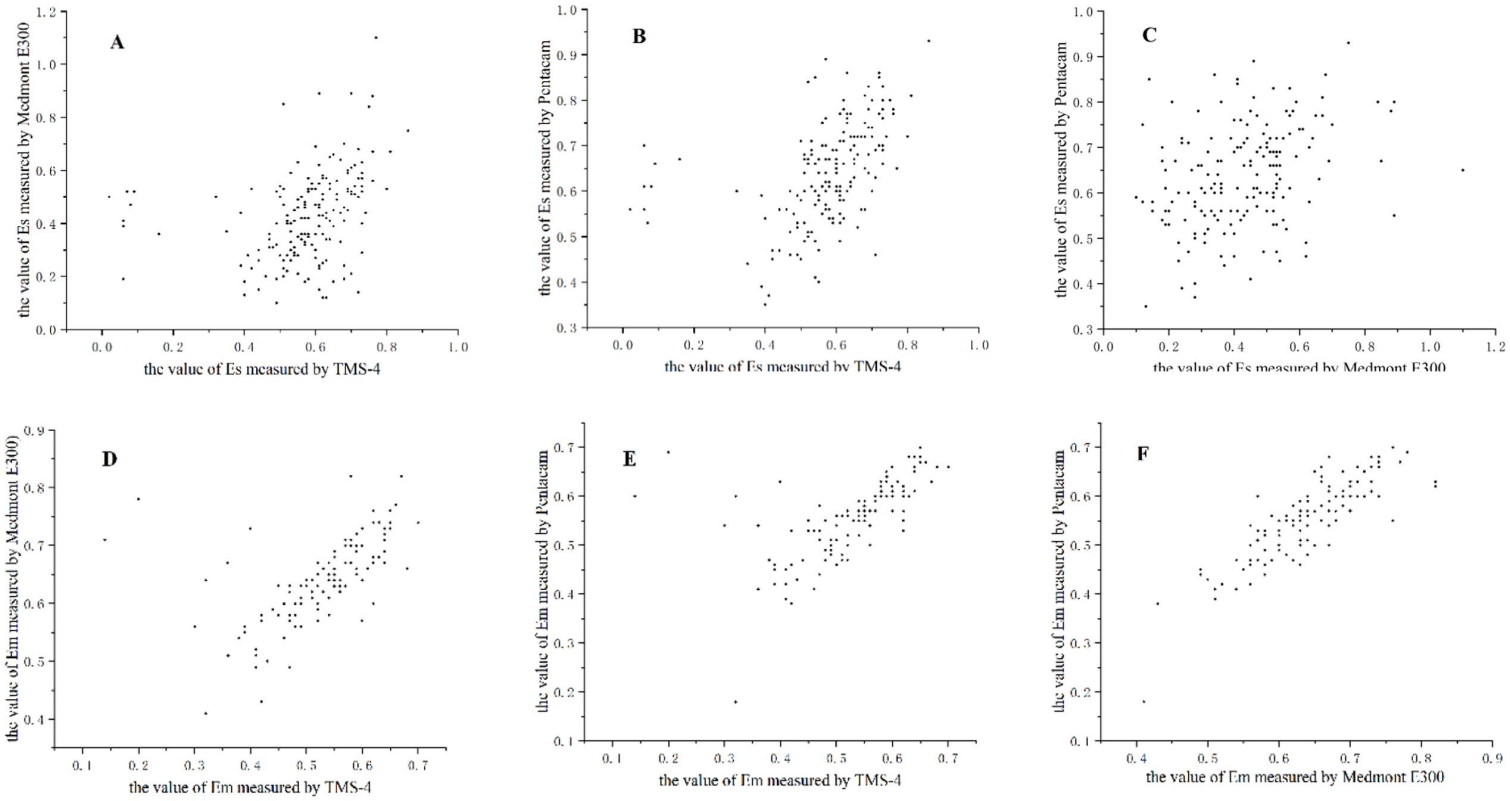
Correlation plots of the corneal eccentricity from theTMS-4 topographer, Medmont E300 topographer and the Pentacam HR for 175 eyes. **(A–C)** Correlation analysis for measurements of steep eccentricity (Es). **(D–F)** Correlation analysis for measurements of flat eccentricity (Em).

**Figure 2:**
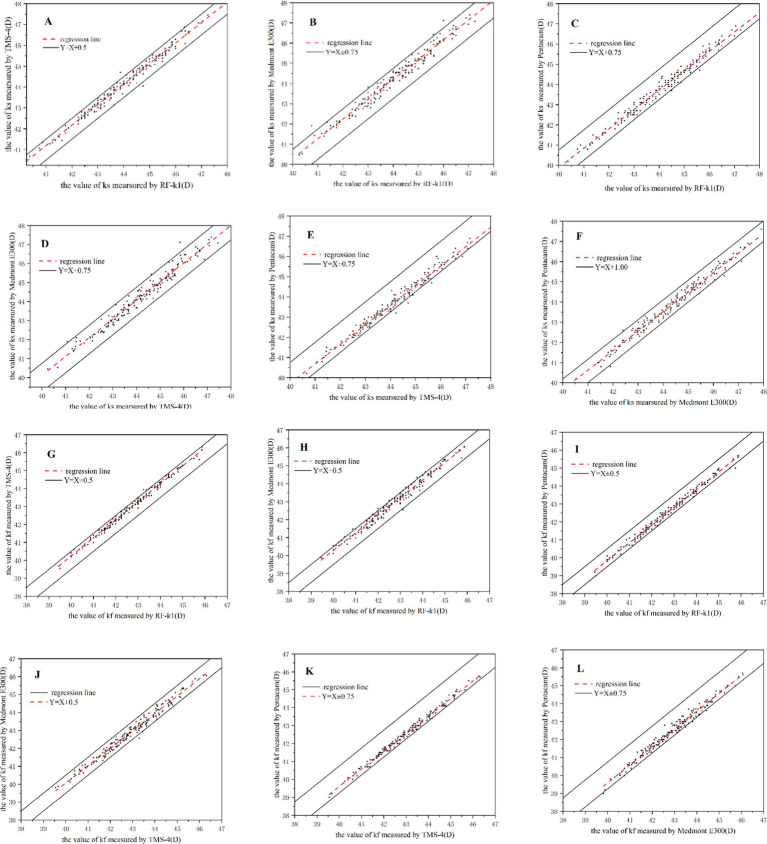
Correlation plots of the corneal curvature from the RK-F1 autorefractors, TMS-4 topographer, Medmont E300 topographer and the Pentacam HR for 175 eyes. **(A–F)** Correlation analysis for measurements of steep corneal curvature (Ks). **(G–L)** Correlation analysis for measurements of flat corneal curvature (Kf).

Comparing the results for eccentricity showed weak correlation and more variability depending on which pairings were assessed. In the comparison of the results for Em, all paired combinations of TMS-4, Medmont E300, and Pentacam HR had strong correlation (*r* = 0.716 ~ 0.838, *p* < 0.01), whereas there was weak correlation between TMS-4 and Pentacam HR in Es (*r* = 0.579, *p* < 0.01). The details are shown in Table 5 and Figures 1, 2.

### Comparison between devices

Table 5 shows the mean difference, SD, and 95% LoA for all paired comparisons of the four devices. The highest mean difference in Ks, Kf, Es, and Em was 0.49D, 0.45D, 0.21, and 0.12, respectively.

As shown in Table 5, there was a statistically significant difference in corneal curvature and eccentricity between any two devices. The highest mean difference of Ks and Kf for all paired comparisons was noted between Medmont E300 and Pentacam HR (0.49 ± 0.21D, *p* < 0.01), and between TMS-4 and Pentacam HR (−0.45 ± 0.13D, *p* < 0.01), respectively. The highest mean difference of Es and Em was also noted between Medmont E300 and Pentacam HR (0.21 ± 0.17, *p* < 0.01) and between TMS-4 and Medmont (0.12 ± 0.09, *p* < 0.01), respectively.

**Figure 3:**
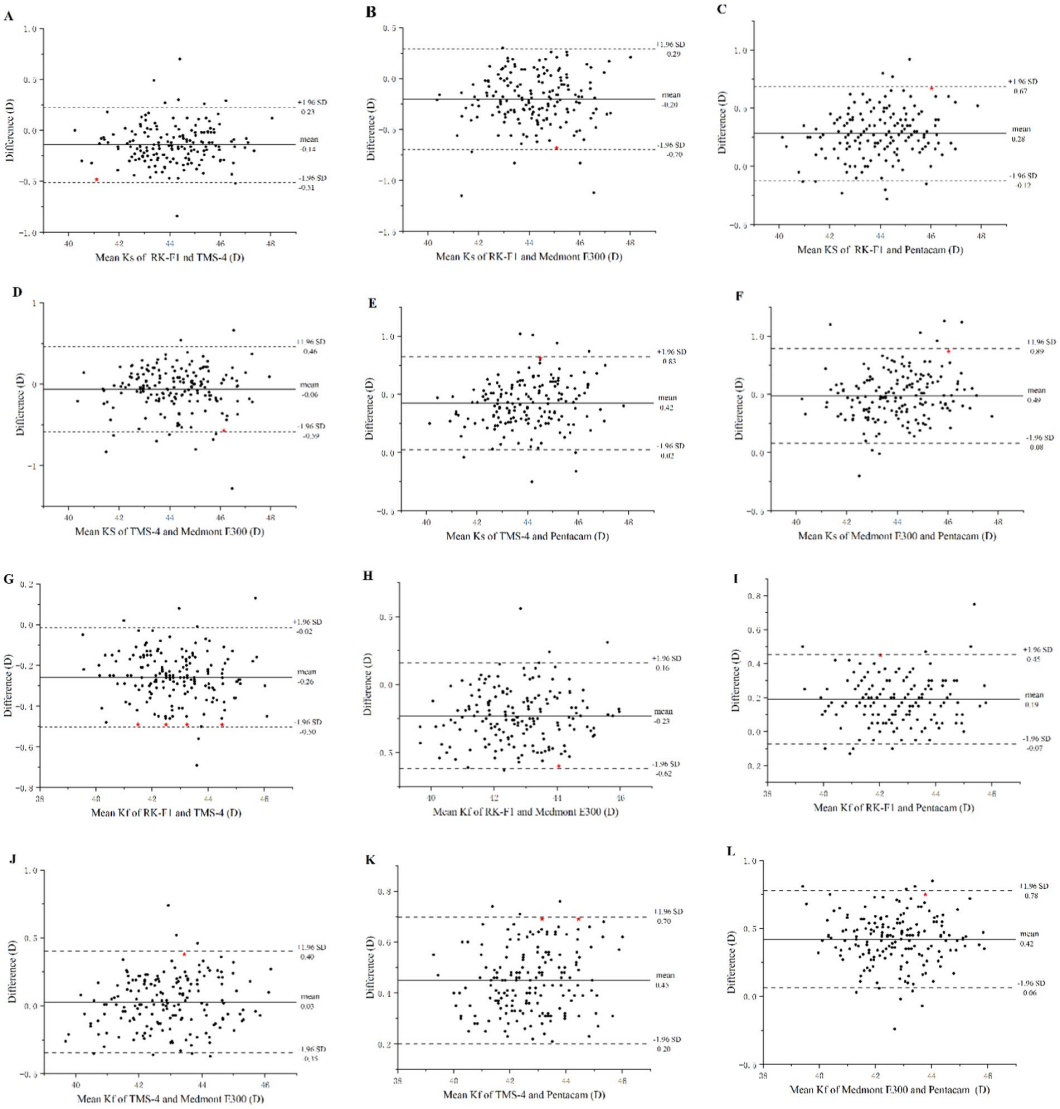
Bland–Altman plots of the corneal curvature from the RK-F1 autorefractors, TMS-4 topographer, Medmont E300 topographer and the Pentacam HR for 175 eyes. **(A–F)** Consistency analysis for measurements of corneal flat curvature (ks). **(G–L)** Consistency analysis for measurements of corneal flat curvature (kf). ★Represents the maximum absolute value of the difference between the measurement results of the two instruments.

**Figure 4:**
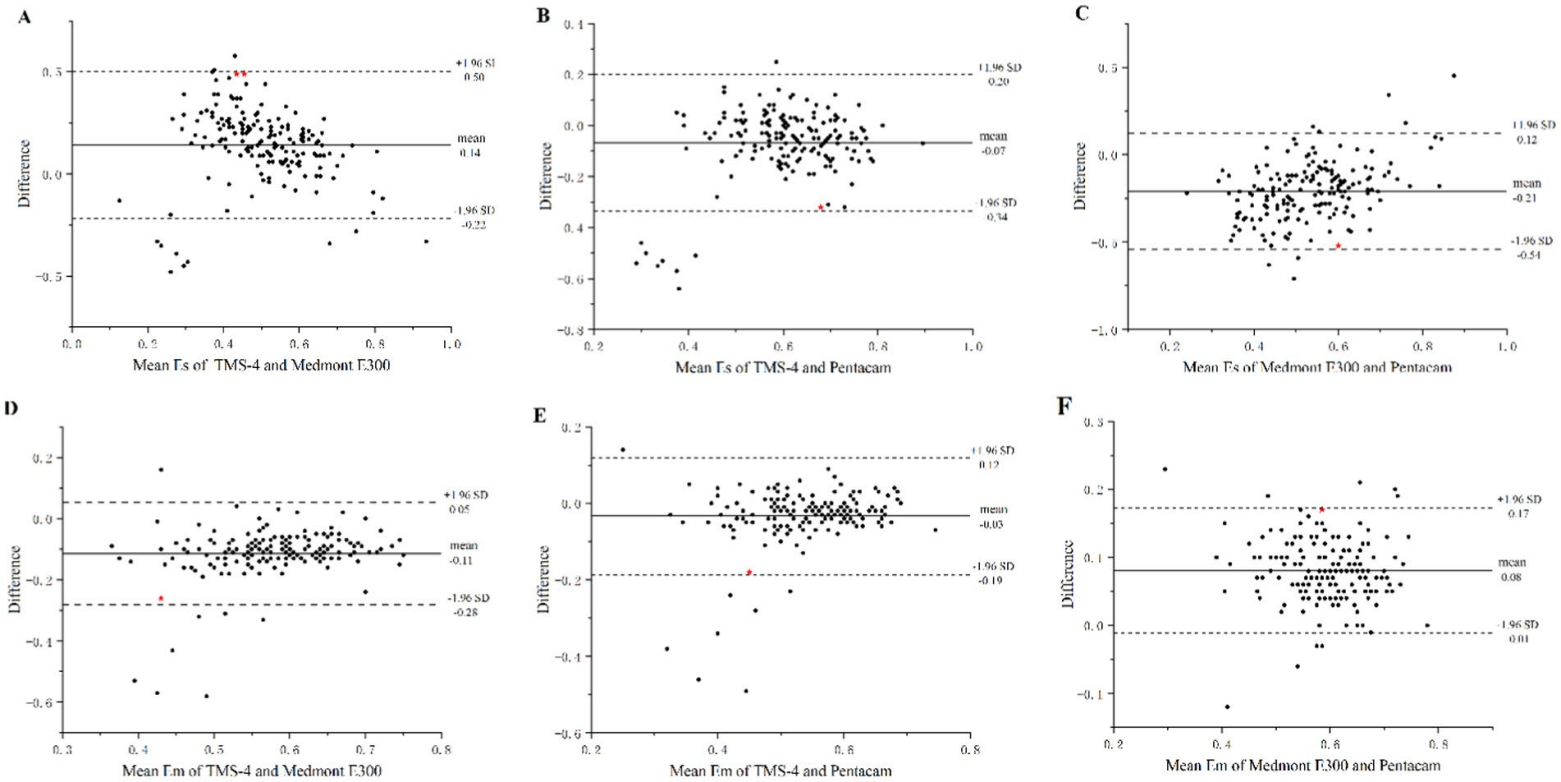
Bland–Altman plots of the corneal eccentricity from the TMS-4 topographer, Medmont E300 topographer and the Pentacam HR for 175 eyes. **(A–C)** Consistency analysis for measurements of steep eccentricity (Es). **(D–F)** Consistency analysis for measurements of corneal flat eccentricity (Em). ★Represents the maximum absolute value of the difference between the measurement results of the two instruments.

Regarding agreement, Table 5 presents the 95% LoA for each pair of devices evaluated. Figures 3, 4 present the corresponding Bland–Altman plots. The 95% LoA for Ks was >0.5D for all paired combinations of the four instruments. Among these paired comparisons, TMS-4 and Medmont E300 showed the largest 95% LoA, ranging from −0.59 to 0.46D. There were 4.57, 3.43, 5.14, 5.71, 4.57, and 4.57% points outside the 95% LoA for all pairings, respectively (Figures 3A–F). Regarding the agreement among the four devices for Kf, the 95% LoA was equal or slightly higher than 0.5D for all paired combinations of all instruments. The RK-F1 and Medmont E300 showed a higher 95% LoA (−0.62 to 0.16D). There were 3.43, 2.86, 4.57, 4, 1.71, and 5.71% points outside the 95% LoA of all pairings, respectively (Figures 3G–L). Regarding the agreement for eccentricity, the 95% LoA for Es was >0.2 for all paired combinations of the instruments, and the TMS-4 and Medmont E300 showed the largest 95% LoA, ranging from −0.22 to 0.50. Regarding the agreement for Em, Medmont E300 and Pentacam HR showed the lowest 95% LoA compared with the other instruments, ranging from 0.01 to 0.17.

## Discussion

The Canon RK-F1 is an autorefractor keratometer that combines the automatic measurement of corneal curvature and refractive power. It can also be used to measure peripheral corneal curvature and corneal diameter. Corneal curvature was measured in the “K-R” model. In the “K-R” model, there is an internal alignment ring composed of solid lines and an external alignment ring composed of small dots on the instrument display screen. The measurement target image is located between the internal and external alignment rings. When the tracking ball is adjusted to align with the pupil center, automatic measurement is started, and each eye is measured three times, respectively (2, 3). The shortage of Canon RK-F1 is it can only measures central 3 mm corneal curvature, and the advantage is rapid and the examination fee is cheap. In optometry clinic, it can help eye doctor quickly screen the ortho-k indications. For example, corneal curvature should be within the range from 39D to 48D. If the corneal curvature is too low or too high, corneal safety risk will increase if wearing ortho-k lens.

Tomey TMS-4 (software version 22C-200S-2A5) is a corneal topographer based on the principle of the Placido disc. The device is equipped with a replaceable 25-ring and 31-ring small cone-disc Placido imaging device, and combined with the computer technology of the color topographic map analysis system, it can obtain images composed of up to 7,936 points in less than 3 s, so as to obtain clear and detailed corneal information (1). The acquired data is processed by a computer and displayed through different topographic map modes.

The Medmont E300 (software version 6.2.7.1) is a Placido disc cone-based computerized videokeratometer, which draws the topographic map of the human corneal surface using a Placido ring. It has 32 Placido rings, covering the cornea in the range of 0.25–11 mm, and analyzes 102,000 points data per scan. A special software was installed in the device that automatically captures the best image based on best positioning, best focus, and minimal eye movement, and scores each picture (100-point scale). A score of 75 or higher was considered good (4–7).

Pentacam HR (software version 1.2r43) is a non-contact anterior segment analyzer with a rotating Scheimpflug camera system. The device uses a 475 nm blue slit light source to illuminate and a red LED light to locate the apex of the cornea. Then, taking the corneal apex as the center, the Scheimpflug camera rotates 360°to take images. Approximately 25–50 Scheimpflug sectional images of the whole cornea can be obtained in 2 s. At least 500 data points are collected for each sectional image, and more than 138,000 data points can be collected for the entire cornea. Then, the computer analyzes these data to obtain the original corneal height data and constructs the 3D model of the anterior segment. In the process of acquiring images, any eye movement is a detected by a second camera and corrected in time (8, 9, 20, 21).

Physicians choose OK lens fitting parameters depending on the Kf and eccentricity values. Accurate measurements of corneal curvature and eccentricity are crucial for OK lens fitting. Kf is the base of selecting alignment curve (AC) parameters of OK lenses, and eccentricity is vital to adjust the AC to be loosen or tighten on cornea to achieve an optimal fitting. Children’s eyes are not comfortable when undergoing the first fitting. If physicians can provide an accurate parameter for children at their first fitting, this may reduce the fitting times and discomfort. In this study, we compared the agreement of corneal curvature and eccentricity measured using four instruments. To the best of our knowledge, no previous study has evaluated the interchangeability and agreement of eccentricity obtained by topographer (TMS-4 and Medmont E300) and anterior segment analyzer (Pentacam HR). Moreover, few studies assessed the agreement of eccentricity on the steep and flat meridians.

The repeatability and reproducibility of the RK-F1 (10), TMS-4 (11), Medmont E300 (12), and Pentacam HR (13) have been confirmed by previous studies. Therefore, this study did not assess the between-examiner and within-examiner differences in corneal curvature and eccentricity measurements. In this study, the results showed that gender and myopia refractive power can affect the accuracy of instrument measurements. Comparing the Ks and Kf in the male group, there was no significant difference among the four instruments, whereas there was a significant difference in the female group. The discrepancy may be related to the sex proportion of subjects, as females accounted for over 60% of the subjects, which was measured more reliably. Comparing the Ks and Kf in different refractive power groups, there was no significant difference in the Kf values for different devices in the mild myopia group, whereas there was a statistically significant difference in Ks and Kf values for all devices in the moderate myopia group. However, the *post hoc* test showed that there was no statistically significant difference between any pair of the four devices. We inferred that this discrepancy may be due to the inherent dependency on the instruments rather than the myopia refractive diopter. Sometimes, patients bring other hospitals’ different topography data to us. If a physician knows the differences between different devices, relatively accurate OK lens alignment curve parameters could be provided to reduce fitting times.

In present study, the measurements of Ks and Kf values obtained by Pentacam HR was the lowest in all instruments. In accordance with our findings, Módis et al. (11) compared TMS-4 with Pentacam HR and found that the latter obtained flatter simulated keratometry values. Other studies have also reported similar results to ours (14, 15). However, Hamer et al. (6) found that the corneal curvature provided by Scheimpflug imaging was steeper than that of Placido disc topography. This discrepancy compared with the current study may be explained by age. In our study, the mean age of all subjects was 10.27 ± 1.99 years, which was far lower than that in Hamer et al.’s study (36.0 ± 11.4 years), and the older subjects may cooperate better than children during the examination. Another study (16) did not find a difference between the Pentacam and Placido disc topographers, but the wide 95% LoA indicated that the measurements cannot be used interchangeably in clinical practice. Regarding the agreement, Pentacam HR showed poor agreement with other devices only on the measurements of Kf between the RK-F1 and Pentacam HR except for, which showed a narrow 95% LoA and small mean difference (−0.07 to 0.45 D, −0.19D, respectively), indicating that the values of Kf obtained by RK-F1 and Pentacam HR can be interchangeable in clinical practice. Tajbakhsh et al. (17) found that Pentacam HR and TMS-4 topography showed the best agreement in Ks and Kf, although the authors used different versions of the Pentacam and different cohorts of patients. In the current study, the highest values of Ks were measured by Medmont E300. This is consistent with previous studies, which reported that Medmont provided a steeper corneal power among the devices (18, 19). Another study (5) assessed the corneal curvature using eight different devices and showed that Medmont E300 had the highest Ks and Kf among all instruments. Therefore, poor agreement between Medmont E300 and other devices was shown in our study. Such comparison data can help physician save time and reduce the ortho-k lens fitting times. Except for OK lens fitting, keratoconus progression or post-corneal collagen cross linking follow-up may undergo different corneal topographers. Our study are helpful to evaluate if cornea curvature is stable. The highest mean differences for Ks and Kf are reported between specific device pairs in our study, which is acceptable and may come from the working principle of the machines and can guide the clinical practice.

For eccentricity, statistically significant differences were found among the TMS-4, Pentacam HR, and Medmont E300 topographers. The Pentacam HR provided the highest Es values, and Medmont E300 provided the highest Em values. The values of Es from TMS-4, Pentacam HR, and Medmont E300 were not in good agreement with each other, whereas the values of Em showed high agreement among the devices. According to the Bland-Alman plots, there were 4.57% (8/175), 4.57% (8/175), and 5.14% (9/175) points outside the 95% LoA among the paired comparison of Em measurements. Cho et al. (4), also reported that the Dicon and Humphrey obtained lower eccentricity compared with Medmont. There was a weak correlation between TMS-4 and Pentacam HR in Es (*r* = 0.579, *p* < 0.01). In optometry, usually physicians choose the first trial lens according to Em or average eccentricity values. Different brand orthokeratology lens have different design, based on the average or flat eccentricity. Eccentricity values are very important in optical clinics.

The main reason for the discrepancy of eccentricity between the different instruments may be due to the different corneal areas selected for analysis. However, in our study, we selected corneas areas within 8 mm except for TMS-4, in which we selected the imaging mode of 25 rings and analyzed the corneal area in the range of 8.8 mm. Therefore, the reason for the lower agreement of Es among the devices is unknown. Physicians usually provide lower alignment curve parameters for higher eccentricity and higher alignment curve parameters for lower eccentricity for proper OK lens fitting. Thus, eccentricity comparison has a clinical significance.

Bland–Altman analysis shows 95% dots were within the range of agreement, which suggest a high level of agreement.

## Conclusion

In conclusion, our data showed that central corneal curvature obtained by RK-K1 autorefractors, TMS-4 topographer, Medmont E300 topographer, and Pentacam HR anterior eye segment analyzer were correlated. Applying central corneal curvature for OK lens fitting is not a good choice. The flat eccentricity obtained among the devices is correlated and comparable, suggesting that the flat eccentricity values could be used interchangeably in clinical practice.

## Data Availability

The original contributions presented in the study are included in the article/supplementary material, further inquiries can be directed to the corresponding author.
